# Tuberculosis treatment outcome among patients treated in public primary healthcare facility, Addis Ababa, Ethiopia: a retrospective study

**DOI:** 10.1186/s13690-020-0393-6

**Published:** 2020-03-10

**Authors:** Atalay Mulu Fentie, Tadesse Jorgi, Tamrat Assefa

**Affiliations:** 1 School of Pharmacy, College of Health Sciences, Addis Ababa University, Addis Ababa, Ethiopia; 2 Addis Ababa Heath Bureau, Addis Ababa, Ethiopia

**Keywords:** Primary health care level, Tuberculosis, Treatment outcomes, Multinomial logistic regression, Adjusted odds ratio

## Abstract

**Background:**

Despite the availability of effective drugs, tuberculosis remains a major public health problem that predominantly affects low- and middle-income countries. This study aimed to assess tuberculosis treatment outcomes among patients treated at one of the primary health care levels in Addis Ababa, Ethiopia.

**Methods:**

An institutional-based retrospective cross-sectional study was conducted at a tuberculosis clinic in public primary healthcare facility. The study populations were all patients with tuberculosis who had been completed their treatment course in the center from July 2014 to July 2018. After getting Ethical clearance and permission from the health center, trained data collectors working in the center were recruited. The collected data were checked for completeness every day by the principal investigators. Data were edited, cleaned, and analyzed using SPSS version 25. Descriptive statistics were used to summarize the data while multinomial logistic regression was employed to explore associations among variables of interest, and *p* < 0.05 was considered as statistically significant.

**Results:**

A total of 352 patients with tuberculosis were included for the study with a median age of 25 years which ranged from 1 to79 year. Most (36.4%) participants were in the age group of 15 to 24 years. The majority (38.8%) of patients had extrapulmonary tuberculosis, 11.9% of them were HIV positive and only two had family history of tuberculosis. Regarding treatment outcome, 238(67.6%) completed the treatment, 95(27%) cured and the rest were unsuccessful treatment outcomes 19(5.4%) either died, defaulted or treatment failed. The odds ratio for cured in relation to unsuccessful treatment outcome was found to be significantly higher in HIV negative patients (AOR = 6.1; 95%CI 2.1–13.9) compared with those patients tested positive for HIV. While patients with smear-positive pulmonary tuberculosis (AOR = 10.5, 95% CI 5.36–16.31) were significantly associated with the odds of having complete treatment cure as compared to patients with extrapulmonary tuberculosis. Similarly being HIV positive and extrapulmonary tuberculosis were predicting factors for unsuccessful treatment compared with their counterparts.

**Conclusions:**

The finding of the present study showed that successful tuberculosis treatment outcome was found to be optimal.

## Introduction

Ethiopia, the 2nd populous country in Africa, is highly affected by tuberculosis (TB) and ranked as 3rd in Africa and 7th among the 22 high TB burdened countries in the world [1]. The prevalence and incidence rate of all forms of TB by 2017 was estimated to be 261 and 359 per 100,000, respectively, and leading to an annual mortality rate of 64 per 100,000 [2].

According to the 2017 Ethiopian Public Health Institute report, the proportion of TB admissions were declining in trend from nearly 5 to 1.5% over the period of 2001/2 to 2015/16 [3]. The TB prevalence and mortality rates were also reduced by > 50% compared to the 1990 baseline values. This might be the fact that considering TB is one of the major public health challenges for the country, the Federal Ministry of Health had given due attention and include TB prevention and control among major priority programs [4]. Directly observed treatment short-course (DOTS) is one of them and was adopted from the WHO with the aim to achieve a 70% case detection rate and 85% treatment success rate. The internationally recommended DOTS strategy for TB control has been recognized as a highly efficient and cost-effective strategy. It comprises five components namely; sustained political and financial commitment, diagnosis by quality ensured sputum-smear microscopy, standardized short-course anti-TB treatment given under direct and supportive observation, a regular, uninterrupted supply of high quality anti-TB drugs and standardized recording and reporting [4, 5]. According to the Federal Ministry of Health assessment in 2013/2014, DOTS program physical coverage reached 98.4% in hospitals and 79% in health centers in the country[3].

Although great efforts are done by different stakeholders; according to the Institute for Health Metrics and Evaluation for Ethiopia, TB is still the 4th most common cause of death [6]. In line with this, in Addis Ababa, of the total 9905 deaths for which the causes were assigned, the proportionate mortality ratio of TB was 6% and higher among males (6.8%) than females (5.1%) [3].

Despite the implementation of DOTS program, different reports across the country have indicated the existence of challenges in improving TB treatment outcomes. The challenges are emanated from differences in treatment-seeking behavior, poor compliance, presence of co-infection, variations in experts’ qualification, and presence of drug resistance [7–13]. Due to these outcome differences, WHO in conjunction with the European Region of the International Union Against Tuberculosis and Lung Disease developed standardized categories to evaluate TB treatment outcomes [11, 14].

Assessing TB treatment outcomes and contributing factors through a continued research can assist policy makers and healthcare providers in planning interventions to overcome the barriers and improve patient treatment response. Moreover, it can serve as an indicator for the quality of TB treatment provided. The present study setting commenced providing service since July 2014 and DOTS program was implemented on the same year. However, TB treatment outcome was not previously studied. Hence, this study aimed to assess TB treatment outcome and associated factors among those patients treated for TB in the primary health care center.

## Methods

The study was conducted in one of the primary health centers found in Kolfe Keranyo subcity, which is one of the densely populated areas in Addis Ababa with a population density of 327 persons per hectare. More than 47,000 patients visit the health center each year. The health center has started and implemented the DOTS program since July 2014.

Institutional based cross-sectional study was conducted and data were collected retrospectively from medical records using a pretested data abstraction form. The data abstraction form was designed to capture TB cases per year, socio-demographic characteristics (sex, age, number of family members, under-5 family members), number of family members screened for TB, family history of TB, comorbid illnesses, type of TB, category of TB treatment, treatment outcome and outcome with respect to time of treatment. All patients treated from July 2014 to July 2018 with complete medical records (either from hospital patient log books or physician/health officer notes on patient charts), who completed their course of treatment or defaulted from their treatment during the study period were included for the study. Patients who were transferred out from the health center were excluded. A total of 357 patients were treated in the health center during the study period. Of these five of them were transferred out and excluded from the study. Ethical clearance and approval of the study protocol were obtained from the Ethical Review Board of Rift Valley University and permission was taken from Kolfe Keranyo Health office and medical director of the health center.

### End points and explanatory variables

According to the definitions and reporting framework of WHO 2013 revision, the following clinical case and treatment outcome definitions were used in this study [14].

#### Case definitions

- *New Case.* A treatment naïve patient or has been on previous anti-TB treatment for < 4 weeks.
- *Re-treatment:* A patient who have received anti-TB drugs ≥1-month in the past [either as *relapse (*a patient declared cured or whose treatment was completed of any form of TB in the past but now found to be smear-positive or culture-positive) or *treatment Failure (*a patient who is smear-positive at the end of 5th months of treatment or later. Treatment failure can also apply for a patient who was initially sputum smear-negative but becomes smear-positive while on treatment) or *return after default (*a patient previously recorded as defaulted from treatment and returns to the health facility with smear-positive sputum)].
- *Transfer-in:* A patient who transferred into the center to finish the course of treatment.

#### Treatment outcome definitions

- *Cured*. Finishing treatment with negative bacteriology results.
- *Completed Treatment*. A TB patient who finished treatment without evidence of failure BUT with no record to show that sputum smear or culture results in the last
  month of treatment and on at least one previous occasion were negative..
- *Treatment Failure*. Remaining smear-positive at 5 months despite the patient took medications correctly.
- *Defaulted treatment*. Patients who interrupted their treatment for ≥2-consecutive months.
- *Death*. Patients who died from any cause during treatment.
- *Unsuccessful treatment outcome*. If TB treatment resulted in treatment failure, defaulted or died. According to definitions and reporting framework of WHO 2013 revision the rest are categorized as successful treatment outcomes [14].

### Data analysis and interpretation

After checking completeness and accuracy, the collected data was entered and analyzed by using SPSS version 25. The collected data completeness and accuracy using data abstraction form was checked by reconciling from source data (both from TB treatment and follow-up log book and patient chart) till zero missed value is obtained. A descriptive statistics were performed and used to summarize socio-demographic data, clinical data and treatment outcome with respect to outcome category time of treatment. Chi-square test was performed to see status of TB treatment outcome with respect to age, HIV status and smear test results. Multinomial logistic regression analysis was conducted to see the association between the dependent (TB treatment outcome) and independent (socio-demographic characteristics, HIV status, type of TB and patient treatment category) variables. In the multinomial logistic regression analysis, “unsuccessful treatment outcome” was the reference category for the dependent variable, which was being compared with the other categories (cure versus unsuccessful treatment outcome; treatment completed versus unsuccessful treatment outcome). All clinically relevant variables and those having at least a marginal association of (*p* < 0.2) in the univariate analysis were included in the multinomial logistic regression analysis to determine potential predictors of unsuccessful treatment outcomes. The *p-*value< 0.05 was considered as statistically significant and both crude and adjusted odds ratio with 95% CI were calculated and presented throughout the result and discussion part. The goodness of fit of model was evaluated by performing chi-square statistics to see whether change in unexplained variance from the baseline model to the final model is significant or not. To say the model is fit, this change must be significant. In addition, Pearson and deviance statistics were also performed to see whether the model is significantly better than no model (*P*-value close to 1 shows the model is fit).

## Results

### Patient characteristics

Among a total of 352 study participants, the majority of them were females 198(56.3%). Regarding age distribution, the median age of study participants was 25 years which ranged from 1 to79 years, with most 128(36.4%) participants were in the age group of 15 to24 years. A lesser 120(34.09%) proportion of participants have voluntarily screened their family members for TB and only 67(19%) of them were living with children < 5 years of age. Only two patients had a family history of TB and 42(11.9%) of them were HIV positive. A greater 133(38.8%) proportion of TB cases were extrapulmonary. Most 321(91.2%) of them were treatment-naive. Only 19(5.4%) of them have visited the health center for re-treatment and 12(3.4%) of them were transferred in from another health facility (Table 1).

**Table 1:** Characteristics of patients treated for tuberculosis at Kolfe, Woreda 11 Health center from July 2014 to July 2018

Variables, *N* = 352	N (%)
Sex	
Male	154 (43.7)
Female	198 (56.3)
Age in years^a^	28.74 ± 12.81, 25 (1,79)
Age category	
≤ 14	19 (5.4)
15–24	128 (36.4)
25–34	113 (32.1)
35–44	50 (14.2)
45–54	19 (5.4)
55–64	14 (4.0)
≥ 65	9 (2.6)
Number of Family members	
≤ 2	81 (52.8)
3–4	108 (30.7)
≥ 5	58 (16.5)
Number of under 5 family members	
0	285 (81.0)
1	54 (15.3)
2	13 (3.7)
Number of family members screened for TB	
≤ 2	202 (57.5)
3–4	97 (27.5)
≥ 5	53 (15)
Family History of TB	
Yes	2 (0.5%)
No	350 (99.5%)
HIV Status	
Positive	42 (11.9)
Negative	281 (79.8)
Unknown	29 (8.2)
Type of Tuberculosis	
Smear positive	95 (27.0)
Smear negative	124 (35.2)
Extra pulmonary	133 (37.8)
Category of patients	
New Case	321 (91.2)
Re-treatment	19 (5.4)
Transferred in	12 (3.4)

^a^*Mean ± SD, Median (Range)*

### Treatment outcomes

From those smears positive 95(27%) patients with TB, majority 60(63.16%) of smear results at 2nd month of treatment were negative. Regarding, smear results at the 5th and 6th months of treatment, only one and no patient had smear-positive result, respectively. About 238(67.6%) patients completed their treatment, 95(27%) cured and the rest of patients 19(5.4%) treatment outcome was unsuccessful. Among those patients whose death reported, majority 11(84.62%) of deaths happened during the continuation phase of treatment (Table 2).

**Table 2:** Treatment outcomes of patients treated for tuberculosis at Kolfe, Woreda 11 Health Center from July 2014 to July 2018

Variables	N	%
Smear result at 2nd month of treatment		
Positive	2	2.1
Negative	60	63.16
Not tested	33	34.74
Smear result at 5th month of treatment		
Positive	1	1
Negative	94	98.99
Not tested	0	0
Smear result at 6th month of treatment		
Positive	0	0
Negative	93	97.89
Not tested	2	2.11
Treatment outcomes		
Cured	95	27
Treatment completed	238	67.6
Treatment failure	2	0.6
Defaulted treatment	4	1.1
Death	13	3.7
Phase of treatment when death reported, *n* = 13		
Initiation	2	15.38
Continuation	11	84.62

The highest 105(29.8%) proportion of TB treatments were given from September 2016 to September 2017. Almost half of 6(46.1%) deaths happened during the first year of DOTS service of the study setting (Table 3).

**Table 3:** Treatment outcomes of patients Treated for Tuberculosis at Kolfe, Woreda 11 Health center from July 2014 to July 2018 with respect to time period

Year	TB treatment outcome					
	Cured N (%)	Treatment completed, N (%)	Defaulted N (%)	Died N (%)	Treatment failed N%	Total N (%)
July, 2014 to July, 2015	17 (17.17)	74 (74.74)	1 (1.01)	6 (6.06)	1 (1.01)	99 (28.12)
August,2015 to August,2016	22 (22.91)	69 (71.87)	2 (2.08)	2 (2.08)	1 (1.04)	96 (27.27)
Sept,2016 to Sept, 2017	35 (33.33)	67 (63.80)	1 (0.95)	2 (1.90)	0 (0)	105 (29.82)
October,2017 to July,2018	21 (40.38)	28 (53.84)	0 (0)	3 (5.76)	0 (0)	52 (14.77)

The TB treatment outcomes of patients with unknown HIV status were either cured (*n* = 6) or completed treatment (*n* = 22). Whereas treatment outcomes with respect to smear test results, except at 5th months (one smear-positive patient’s treatment failed), all unsuccessful treatment outcomes were in those patients whose smear test was not done either they were diagnosed with extrapulmonary TB, smear-negative at time of diagnosis or not tested because two patients who were tested positive at 5th months of treatment have died (Table 4)

**Table 4:** Status of TB treatment outcome with respect to age group, HIV status and smear test results

Variables	Cured	Completed treatment	Failure	Defaulted	Death	*p*-value
Age in years						0.117
≤ 24, *n* = 147	36 (24.5)	108 (73.5)	0 (0)	1 (0.7)	2 (1.4)	
25–34, *n* = 113	35 (30.9)	72 (63.7)	1 (0.9)	1 (0.9)	4 (3.5)	
35–44, *n* = 50	16 (32.0)	28 (56.0)	1 (2.0)	2 (8.0)	3 (6.0)	
45–54, *n* = 19	4 (21.1)	12 (63.2)	0 (0)	0 (0)	3 (15.8)	
≥ 55, *n* = 23	4 (17.4)	18 (78.3)	0 (0)	0 (0)	1 (4.3)	
HIV status						0.000
Positive, *n* = 42	10 (23.8)	21 (50.0)	1 (2.4)	2 (4.8)	8 (19.0)	
Negative, *n* = 281	79 (28.1)	194 (69.0)	1 (0.4)	2 (0.7)	5 (1.8)	
Unknown, *n* = 29	6 (20.7)	22 (75.9)	1 (3.4)	0	0	
Smear result at 2nd month of treatment						0.000
Positive, *n* = 2	1 (50.0)	1 (50.0)	0	0	0	
Negative, *n* = 60	60 (100)	0	0	0	0	
Not tested^a^, *n* = 290	34 (11.7)	237 (81.7)	2 (0.7)	4 (0.14)	13 (4.5)	
Smear result at 5th month of treatment						0.000
Positive, *n* = 1	0	0	1 (100)	0	0	
Negative, *n* = 92	92 (100)	0	0	0	0	
Not tested^a^, *n* = 259	3 (1.2)	237 (91.5)	2 (0.8)	4 (1.5)	13 (5.0)	
Smear result at 6th month of treatment						0.000
Positive, *n* = 0	0	0	0	0	0	
Negative, *n* = 92	92 (100)	0	0	0	0	
Not tested ^a^, *n* = 260	3 (1.6)	238 (91.5)	2 (0.8)	4 (1.6)	13 (5.0)^b^	

Chi-square test result, ^a^All patients with extrapulmonary TB, smear negative at time of diagnosis and not tested despite they were smear-positive at time of diagnosis. ^b^two smear-positive patients who have positive-smear test result at 5th months not tested is because they were died.

Majority 333(94.6%) of TB treatment outcomes were successful i.e. either cured or successfully completed their treatment, whereas only 19(5.4%) of treatments were unsuccessful either defaulted for treatment (*n* = 4), treatment failure (*n* = 2) or died (*n* = 13).

### Predictive factors for tuberculosis unsuccessful treatment outcome

To assess possible predicting factors affecting treatment outcome, all clinically relevant and variables that showed marginal association at *p* < 0.2 following univariate analysis were included in the multinomial logistic regression analysis. Of those four variables which fulfilled the inclusion criteria for the multinomial logistic regression analysis; only type of TB and HIV status were significantly associated with treatment success rate.

The odds of cured was significantly higher in HIV-negative patients (AOR = 6.1; 95%CI 2.1–13.9, *p = 0.045*) compared with those patients tested positive for HIV. Besides, patients with smear-positive pulmonary TB (AOR = 10.5, 95% CI 5.36–16.31, *p = 0.003*) were more likely to be cured as compared to patients with extrapulmonary TB. Regarding the comparison of treatment completed vs unsuccessful treatment outcomes; the odds of completing treatment successfully among patients tested negative for HIV were 4.4(AOR = 4.4, 95% CI: 1.26–15.58, *p* < 0001) times more likely compared with their counterparts. Similarly, the odds of completing treatment successfully was found to be higher in patients with smear-positive pulmonary TB (AOR = 3.8;95%CI:1.25–7.4;,*p = 0.01*) than patients with extrapulmonary TB.

The change in unexplained variance from the baseline model (−2LL = 413.5) to the final model (−2LL =48.7) was significant (Model X^2^ = 364.7). This change is significant, which means that our final model explains a significant amount of the original variability (in other words, it’s a better fit than the original model). In addition, Pearson and deviance statistics were also much higher with a respective *p*-value of (*p* = 0.730) and (*p* = 0.894), which means the model is significantly better than no model (i.e the model is good fit of the data). Besides overdispersion is not the problem for the model since *p* = 0.730 and *p* = 0.894 are much higher than 0.05 (Table 5).

**Table 5:** Multinomial regression of predictive factors associated with treatment success rate among patients treated for TB

			COR (95% CI)		AOR (95% CI)
Cured Vs unsuccessful treatment outcome		B(SE)		B(SE)	
Age category, in years		−0.02 (0.02)	0.97 (0.94–0.0.99)*	− 0.04 (0.05)	0.91 (0.71–2.33)
HIV status	Positive		1.00		1.00
	Negative	2.2 (0.35)	8.78 (4.41–17.51)**	2.34 (1.8)	6.1 (2.1–13.9)*
	Unknown	1.8 (1.1)	6.00 (0.72–13.1)	0.64 (1.68)	1.9 (0.07–51.4)
Type of TB	Extra PTB		1.00		1.00
	Smear positive PTB	2.8 (0.46)	17.8 (7.2–43.8)**	4.66 (1.2)	10.5 (5.36–16.31)**
	Smear negative PTB	−1.4 (0.65)	0.25 (0.071–0.89)*	− 0.27 (1.1)	0.77 (0.09–6.58)
TB treatment category	New		1.00		1.00
	Retreatment	−1.6 (0.27)	0.52 (0.34–0.86)*	− 1.1 (0.22)	0.31 (0.05–0.59)
	Transfer in	0.85 (0.69)	2.33 (0.60–9.02)	0.54 (0.39)	2.01 (0.87–3.65)
Treatment completed Vs unsuccessful treatment outcome					
Age category in years		−0.03 (0.02)	0.97 (0.93–0.95)*	− 0.016 (0.81)	0.99 (0.81–2.99)
HIV status	Positive		1.00		1.00
	Negative	3.1 (0.34)	21.4 (10.9–39.8)**	2.07 (1.13)	4.4 (1.26–15.58)*
	Unknown	0.65 (0.0.37)	1.9 (0.92–3.91)	0.18 (1.1)	2.84 (0.096–7.3)
Type of TB	Extra PTB		1.00		1.00
	Smear positive PTB	2.2 (0.31)	9.1 (5.00–16.5)**	4.26 (1.28)	3.8 (1.25–7.4)**
	Smear negative PTB	3.46 (0.51)	2.88 (1.17–7.39)**	0.91 (0.62)	1.73 (0.12–4.34)
TB treatment category	New		1.00		1.00
	Re-treatment	−1.3 (0.67)	0.273 (0.024–0.81)*	− 1.9 (0.86)	0.15 (0.03–0.99)
	Transfer in	0.11 (1.04)	1.42 (0.62–7.2)	3.2 (1.65)	2.11 (0.65–3.88)

*Note*: *R*^2^ = 0.645 (Cox & Snell), 0.813 (Nagelkerke); Model *χ*2 = 364.7, Pearson (*p* = 0.73) and deviance(*p* = 0.894); * *p* < .05, ** *p* < .001

## Discussion

Assessment of TB treatment outcome, as well as analysis of factors responsible for poor treatment outcome, is one of the major indicator for the evaluation of the performance of a national TB program. Our study found that the treatment outcome of patients with TB treated under DOTS program at Kolfe, Woreda 11 health center was satisfactory (94.6% successful treatment outcome rate). Type of TB, HIV status of patients and TB treatment category were significantly associated with treatment success rate.

The successful treatment outcome (94.6%) in this study is above our national millennium goal (85%) and in line with the WHO target(> 90%) [4, 5], a study done at Enfraz health center, Northwest Ethiopia (94.8%) [15] and Turkey (92.6%) [16]. In contrary, according to the meta-analysis done by Eshetie et al (83.7%) [9], still, the Ethiopian TB treatment success rate is lower than the national goal (85%) and WHO target level (> 90%). In addition lowest successful TB treatment outcomes were reported in two subsequent studies done in University of Gondar Teaching Hospital for the year 2008–2012 (60.1%) [17] and among HIV/TB co-infected patients (77.3%) [18] and other studies done at Metema Hospital (65.3%) [19], Felege Hiwot Hospital (80.8%) [20], Jinka General Hospital (74%) [21] and Asella Teaching Hospital (81.7%) [22]. This variation might be due to difference in the quality of DOTS service and study area where the present study was done among urban patients compared to others where more rural community with little knowledge about TB, lower information about their treatment, inappropriate health-seeking behavior and stigma towards TB [12, 22]. This was also supported by a small number of transfer outpatients (*n* = 5) and treatment defaulters (*n* = 4) in the present study.

Apart from Ethiopia, the lowest treatment success rates were also reported in Nigeria (81.4%) [23], a meta-analysis done in India (39%) [24] and in Europe as well (70.7%) [25]. The lowest success rate report in Europe might be due to immigration to Europe from Africa and Asia, affecting treatment processes.

During the study period, out of 352 patients with TB who were registered at the health center for treatment; 67.6% completed their treatment, 27% cured, 3.7% died, 1.1% defaulted and 0.6% failed the treatment regimen. The Study finding is comparable with a study done in Debre Tabor were among a total of 985 patients, 90.1% of them had successful treatment outcomes (cured and treatment completed), while 74 patients (9.9%) had unsuccessful treatment outcomes (death and treatment failure) [26]. But in other study done at Gondar from 2008 to 2012, significantly differed treatment outcomes were reported such as 46.4% completed treatment, 13.7% cured, 17.7% died, 21.3% defaulted and 0.8% had treatment failure [17]. This might be due to difference in the study participants with respect to literacy level, distance between patients’ home and health care center that present study participants live only in capital city of Ethiopia. Both may lead the patient to be diagnosed at more advanced stage of the disease and also significantly affect patients’ adherence to treatment.

In this study multinomial logistic regression analysis indicated that probability of cure compared to unsuccessful treatment outcome in patients with smear-positive pulmonary TB (AOR =10.5, 95% CI 5.36–16.31, *p = 0.003*) and HIV-negative (AOR = 6.1; 95%CI 2.1–13.9, *p = 0.045*) were significantly associated with the odds of increased cure as compared to extrapulmonary TB and patients with HIV co-infection, respectively. In addition, completing treatment successfully with respect to unsuccessful treatment outcome, the odds of completing treatment successfully was higher in patients with smear positive pulmonary TB and those tested negative for HIV, compared with their counterparts. In contrary, according to a study done in Nigist Eleni Mohammod General Hospital Hosanna, Ethiopia; those patients having smear-positive Pulmonary TB were more likely to had poor treatment outcomes compared with those patients having extra-pulmonary TB [7]. But, the other studies done in other parts of Ethiopia [15, 20] and China [27] reported as there is no significant difference between treatment success rate and type of TB. According to studies conducted at Jinka General Hospital [21], Afar [28] and another study done by Kefale et al at Southwest Ethiopia [29]; TB/HIV co-infection was found to be a predictor for unsuccessful treatment outcome, which is similar to the present study.

In the present study, there was no statistically significant association between patients with TB who came for re-treatment and TB-treatment naïve patients. However, according to a study conducted at Hawassa, being on re-treatment, smear-positive after 2-months of treatment were reported as predictors of unsuccessful treatment outcome [22]. In addition, according to a study done in India, re-treatment was found to be a predictor for unsuccessful treatment outcome [30].

In this study, though treatment outcome as per WHO definition is found to be good, the use of retrospective secondary data may affect the success rate because it was totally dependent on the documented data in the TB DOTS registration logbook and cannot control the outcome. As we didn’t have available smear test results, the vast majority of successful cases (70.2%) were categorized as “treatment completed” and which is actually reported as risk for treatment relapse [31, 32]. Being a single-center study, the results may not be generalized to all other health centers situated in Addis Ababa. Excluding transfer out patients from the present study may also be a source of possible bias and affect generalizability to the study population. The excluded five transferred out patients may also positively or negatively affect the treatment success rate. Being unsuccessful treatment outcome is small [19(5.4%)], the effect of the transfer out patients might be significant on the outcome evaluation. If all the transfer out patients treatment outcome is categorized in the unsuccessful treatment outcome wing, then percentage of unsuccessful treatment outcome becomes 6.7% or increased by more than 20%(unsuccessful treatment outcome = 19 + 5 = 24; = 24/ (352 + 5) = 6.7%). Despite these limitations, we believe that the study provides baseline information about treatment outcome and associated factors which can be helpful for healthcare providers working in the center and other policy makers for further intervention. It can also be helpful to conduct further research in the same or other centers.

## Conclusion

Tuberculosis treatment success rate at Woreda 11 health center was in general good compared to other studies conducted in Ethiopia and is above the target set by WHO and our national TB and leprosy control program. Those patients with HIV and having extra-pulmonary TB were predictors for unsuccessful treatment outcome compared with HIV negative and smear positive pulmonary TB, respectively.

## Data Availability

All the data and materials used in this paper will be available upon request.

## Abbreviations

AOR: Adjusted odds ratio
COR: Crude odds ratio
DOTs: Direct observed treatment strategies
HIV: Human immune deficiency virus
SPSS: Statistical package for social science
TB: Tuberculosis
WHO: World Health Organization
